# Reviewed article: Filho ZVN, Silva NC, Fé RASS, Lourenço MAG, Pazinatto RB, Cabral AEA, Melo LA. Lifestyles associated with complete tooth loss in elderly people in Brazil, 2019. Epidemiol Serv Saude. 2025:34;e20240614

**DOI:** 10.1590/S2237-96222025v34e20240614.a

**Published:** 2025-05-12

**Authors:** Leandro Aparecido Souza

**Affiliations:** UNISO

**Table:** 

Rating	Excellent	Good	Average	Below average	Poor
Interest		✓			
Quality		✓			
Originality		✓			
Overall		✓			

**Table:** 

Questionnaire	Yes	No	Not applicable
Does the manuscript contain new and significant information to justify publication?	✓		
Does the Abstract (Summary) clearly and accurately describe the content of the article?	✓		
Is the problem significant and concisely stated?		✓	
Are the methods described comprehensively?	✓		
Are the interpretations and conclusions justified by the results?	✓		
Is adequate reference made to other work in the field?		✓	

## Manuscript Structure:

Length of article is: Adequate

Number of tables is: Adequate

Number of figures is: Adequate

Please state any conflict(s) of interest that you have in relation to the review of this paper (state “none” if this is not applicable): None

